# Transcriptome profiling analysis reveals key genes of different coat color in sheep skin

**DOI:** 10.7717/peerj.8077

**Published:** 2019-11-21

**Authors:** Lidan Yao, Aodungerile Bao, Wenjuan Hong, Chenxi Hou, Zhenliang Zhang, Xiaopeng Liang, Jueken Aniwashi

**Affiliations:** College of Animal Science, Xinjiang Agricultural University, Urumqi, China

**Keywords:** Sheep, RNA-seq, Molecular mechanism, Coat color

## Abstract

**Background:**

To investigate the molecular mechanisms determining the coat color of native breed sheep in Xinjiang.

**Methods:**

Bashibai sheep, Yemule white sheep and Tulufan black sheep were selected. Illumina HiSeq X Ten sequencing technology was used to detect the genes responsible for the white, light brown, black and cyan gray coat colors in sheep. Sequence analysis and functional gene annotation analysis were performed to analyze the results. The signal pathways and differentially expressed genes related to sheep hair color production regulation were screened and finally verified by real-time polymerase chain reaction.

**Results:**

Functional annotation by Kyoto Encyclopedia of Genes and Genomes analysis revealed significant differences in enrichment of immunity-related pathways as well as melanogenesis synthetic and tyrosine metabolism pathways. Our results showed that the *DCT*, *TYR*, *TYRP1*, *PMEL*, *SLC45A2* and *MLANA* six genes may be associated with the regulation of coat color development and provide a theoretical basis for selecting natural coat colors of sheep.

## Introduction

The sheep were one of the earliest domesticated animals worldwide and the wool of sheep is an important textile material. Chinese sheep colors mainly include red brown, yellow brown, black, white, spotted and other colors, which are determined by both genetics and the environment. In several vertebrates, the colors of the eyes, feathers and skin are primarily determined by the amount and distribution of melanin, which is secreted by mature melanocytes at the base of the epithelium (Simon et al., 2009; Cieslak et al., 2011). There are two types of melanin: pheomelanin, which is a round red particle dissolved in alkali environments and causes a yellow and red coat color and eumelanin, including black and brown pigment types, the ratio of which determines the animal coat color phenotype (Yue et al., 2015; Visscher, 2017). Melanocytes are derived from the embryonic neural crest and melanoblast specification and migration as well as melanocyte differentiation depend on the regulation of different genes. After the binding of melanocortin one receptor (*MC1R*) on the cell membrane to melatonin (α-MSH) and adrenocorticotropic hormone, the coupled G protein is converted to GTP, thereby activating the adenylate ring on the membrane. The enzyme system produces cyclic adenosine monophosphate (cAMP) and further activates the tyrosine (*TYR*) kinase, which, in turn, activates *TYR* to participate in melanin synthesis. Under normal conditions, melanocytes synthesize pheomelanin; otherwise, under conditions of excessive activation of *TYR*, they synthesize eumelanin (Abdel-Malek et al., 1995; Suzuki et al., 1996; Marklund et al., 1996). *MC1R* is antagonized by agouti signaling protein (*ASIP*), which competes with α-MSH for binding to *MC1R*. *ASIP* binds to *MC1R* to block the initiation signal of α-MSH, which blocks the production of cAMP, ultimately leading to increased synthesis of eumelanin and decreased synthesis of pheomelanin (Jackson et al., 2006).

The number of white sheep is the largest in the world as their coat can be dyed to virtually any color. In Xinjiang, the local sheep have four coat colors. Previous studies have identified two alleles at the extension locus in sheep: the dominant black allele (E^D^), which accounts for the black pigmentation of colored breeds and the wild type allele (E^+^), which is widely distributed in most breeds (Våge et al., 1999; Yang et al., 2013; Mahmoud et al., 2017) and has also been observed in 13 breeds of sheep in China (Yang et al., 2013). Significant associations between these alleles and coat color were found within or near the *ASIP*, *ITCH*, *AHCY* and *RALY* genes on chromosome 13 for black and brown coat colors and the *KIT* and *PDGFRA* genes on chromosome six for white coat color using genome-wide association studies in Markhoz (Nazari-Ghadikolaei et al., 2018). A regulatory mutation identified in the *ASIP* gene was associated with the black recessive non-agouti (Aa) allele (Norris & Whan, 2008; Gratten et al., 2010). In addition, two mutations in *ASIP* (recessive black allele: g.100_105del (D_5_) and/or g.5172T>A) were associated with black coat color in Klövsjö and Roslag sheep breeds and in *MC1R* (dominant black allele: c.218T>A and/or c.361G>A) were associated with black coat color in Swedish Finewool sheep (Rochus et al., 2019).

There are approximately 26 million sheep in Xinjiang, China, with rich and varied coat colors, some of which have four or more coat color phenotypes. Local varieties mostly have coarse wool, which is difficult to utilize and thus, these sheep are mostly used for meat production. However, Xinjiang local sheep produce 1.2–1.9 kg of wool per year per sheep, with a cashmere content of 55.5–58.5%, which can be used as a textile material. Based on the cashmere fitness and length, the four phenotypes of the coat are in the order of light brown > black > cyan gray > white, with cashmere fineness of 20.94 ± 4.03 μm and length of 14.03 ± 1.4 cm for light brown; 20.45 ± 4.13 μm and 20.2 ± 1.6 cm for black; 21.49 ± 4.40 μm and 15.2 ± 1.8 cm for cyan gray; and 21.21 ± 4.51 μm and 19.14 ± 2.6 cm for white. Therefore, understanding the regulation mechanism of wool color determination of local breeds of sheep in Xinjiang is important. In this study, four types of wool color phenotypes observed in sheep in Xinjiang were conducted transcriptome sequencing to analyze the differentially expressed genes. These results provide molecular theoretical basis for breeding sheep with different wool colors.

## Materials and Methods

Thirty-six healthy 1-year-old white (Yemule white sheep), black (Tulufan black sheep), light brown and cyan gray (Bashibai sheep) ewe (nine sheep per color) were selected for sample collection from a sheep farm in Xinjiang. Local anesthesia to decrease animal suffering was performed with procaine hydrochloride (1.5 mL, 3%) after obtaining approval (reference number 2018004) to conduct the study from the Animal Hospital of Xinjiang Agricultural University. Two pieces of skin (10 mm in diameter) from the scapular area were collected and immediately placed into liquid nitrogen. Three sheep skin tissues of each color were subjected to sequencing. Light brown skin sample numbers were R1, R2 and R3; black skin sample numbers were B1, B2 and B3; cyan gray skin sample numbers were G1, G2 and G3; and white skin sample numbers were W1, W2 and W3. Total RNA from samples was extracted using Trizol reagent (Invitrogen, Carlsbad, CA, USA) according to the manufacturer’s instructions. Sequencing libraries were generated using the NEBNext Ultra™ RNA Library Prep Kit for Illumina (New England Biolabs, Ipswich, MA, USA) following the manufacturer’s recommendations and index codes were added to attribute sequences to each sample. To select cDNA fragments of approximately 240 base pairs in length, the library fragments were purified with the AMPure XP system (Beckman Coulter, Brea, CA, USA). Next 3 μL USER Enzyme (New England Biolabs) was added to size-selected, adaptor-ligated cDNA at 37 °C for 15 min followed by 5 min at 95 °C before PCR.

### Quality control of sequencing data

Library sequencing was performed using the Illumina HiSeq X Ten (San Diego, CA, USA). The raw data file obtained by high-throughput sequencing was transformed into the original sequencing data after base recognition analysis. Fast QC software was used for evaluating sequenceing quality (QC) and then the reads containing adapters, high N contents, or numerous low-quality bases were filtered out to obtain clean reads. Hisat2 tools were used to map the reads to the reference genome (Nazari-Ghadikolaei et al., 2018). StringTie (Pertea et al., 2015) was used to splice the alignment results of Hisat2 and the type of alternative splicing and the corresponding expression amount of each sample were obtained by ASprofile (Florea, Song & Salzberg, 2013) software. The sequencing data of 12 samples were submitted to the Sequence Read Archive (Black group: SRR10143190, SRR10143189, SRR10143186, Cyan gray group: SRR10143185, SRR10143184, SRR10143183, Light brown group: SRR10143182, SRR10143181, SRR10143180, White group: SRR10143188, SRR10143187, SRR10143179) in NCBI.

### Differential expression analysis

Transcription levels were calculated using the fragments per kilobase per million mapped reads (FPKM) method. Using DESeq (Love, Huber & Anders, 2014) software to analyze the differential expression between sample groups, two different expression gene sets between biological conditions were obtained and a fold-change ≥2 and false discovery rate <0.05 were set as the threshold for significant differential expression. The fold-change indicates the ratio of the expression levels between the two groups. DESeq provide statistical routines for determining differential expression in digital gene expression data using a model based on the negative binomial distribution. The resulting *P* values were adjusted using the Benjamini and Hochberg’s approach for controlling the false discovery rate. Genes with an adjusted *P*-value < 0.05 found by DESeq were assigned as differentially expressed.

### Gene function annotation

Gene function was annotated using the following databases: NCBI non-redundant protein sequences (Nr) (Deng et al., 2006), NCBI non-redundant nucleotide sequences (Nt), Protein family (Pfam) (Finn et al., 2014), KOG, Clusters of Orthologous Groups of proteins (COG) (Tatusov et al., 2000; Koonin et al., 2004), Swiss-Prot (A manually annotated and reviewed protein sequence database), Kyoto Encyclopedia of Genes and Genomes (KEGG Ortholog database) and Gene Ontology (GO) (Ashburner et al., 2000).

### Quantitative real-time PCR validation

Differentially expressed genes related to coat color were selected. Referring to the sequences of these genes in sheep published in GenBank, specific primers were designed using Primer Premier 5.0 software and synthesized by Bioengineering (Shanghai) Co. Ltd. (Table S1). The relative expression of genes was statistically analyzed by the 2^−ΔΔCt^ method, statistical analysis was performed using student’s *t*-test (*p < 0.05, **p < 0.01).

## Results

### Data quality control

The base content and base mass distribution statistics of clean reads were obtained by quality control of sequencing data (Figs. S1 and S2). The effective GC ratio was above 51%. Quality value Q 30 means that the base recognition accuracy rate is 99.9%, which is above 94% and that the ratio of each sample aligned to reference genome was 89.69–90.29% (Table S2). Additionally, most read were mapped to the EXON region (Fig. S3), which overall indicates good sequencing data and high utilization. A total of 12 types of variable shear were found in the results of variable splicing analysis, the most important of which were: variable 1st exon (alternative first exons, TSS), variable end exon (alternative last exons, TTS), single exon jumps (skipped exons, SKIP), and variable 5′ or 3′ end shear (alternative exon ends 5′, 3′, or both).

### Analysis of differentially expressed genes

**Figure 1:**
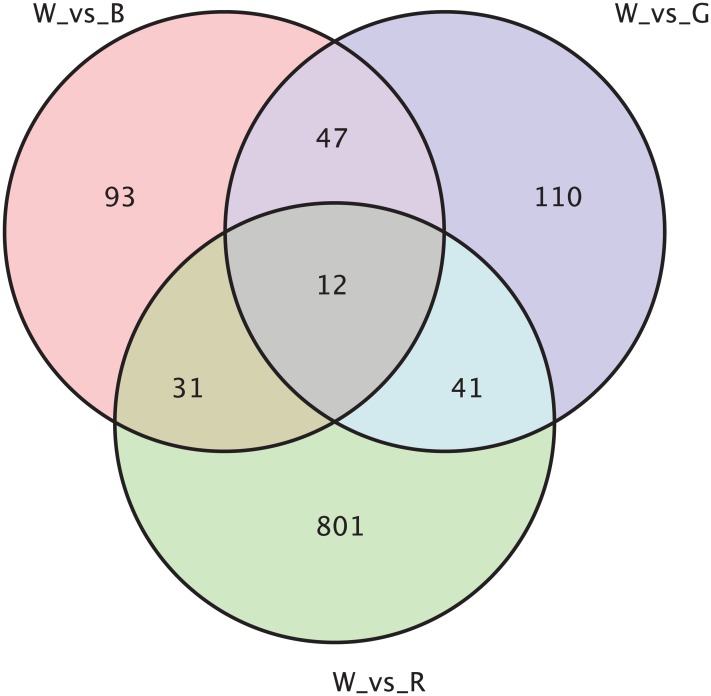
Venn diagram of differentially expressed genes.

According to the level of gene expression (FPKM), a fold-change ≥2 and false discover rate <0.05 were used as screening criteria. The FPKM box plot of each sample was drawn (Fig. S4) and the gene expression levels of individual samples were distributed at the same expression level. The overall expression levels of the samples were also nearly identical. Additionally, the clustering maps of differentially expressed genes in each group were drawn (Fig. S5). Each group of samples was clustered together and three samples from the same group were clustered in some cluster. Sequence alignments were performed using the NR, GO, COG, KOG and KEGG databases and differences in gene expression among the four color phenotype groups were calculated (Table 1). The white sheepskin was used as the control group and the other three were used as comparison groups (W_vs_B, W_vs_G, W_vs_R). A total of 183 significantly differentially expressed genes in the white and black groups, and 91 up-regulated genes and 92 down-regulated genes in the black group were observed. There were 210 significantly differentially expressed genes in the white and cyan gray group, 56 up-regulated genes and 154 down-regulated genes in the cyan gray group, 885 differentially expressed genes in the white and light brown groups, 584 up-regulated genes and 301 down-regulated genes in the light brown group. Additionally, the number of differentially expressed genes in the three groups was counted and plotted as a Venn diagram to show the number of unique and differentially expressed genes between groups (Fig. 1).

**Table 1:** Differentially expressed genes annotation by searching against public databases.

Group	NR	COG	KOG	GO	KEGG	DEGs-total	DEGs-up	DEGs-down
W_vs_B	179	55	82	147	118	183	91	92
W_vs_G	209	56	97	176	154	210	56	154
W_vs_R	851	207	411	661	558	885	584	301

### GO, COG, KOG and KEGG enrichment analysis of differentially expressed genes

**Figure 2:**
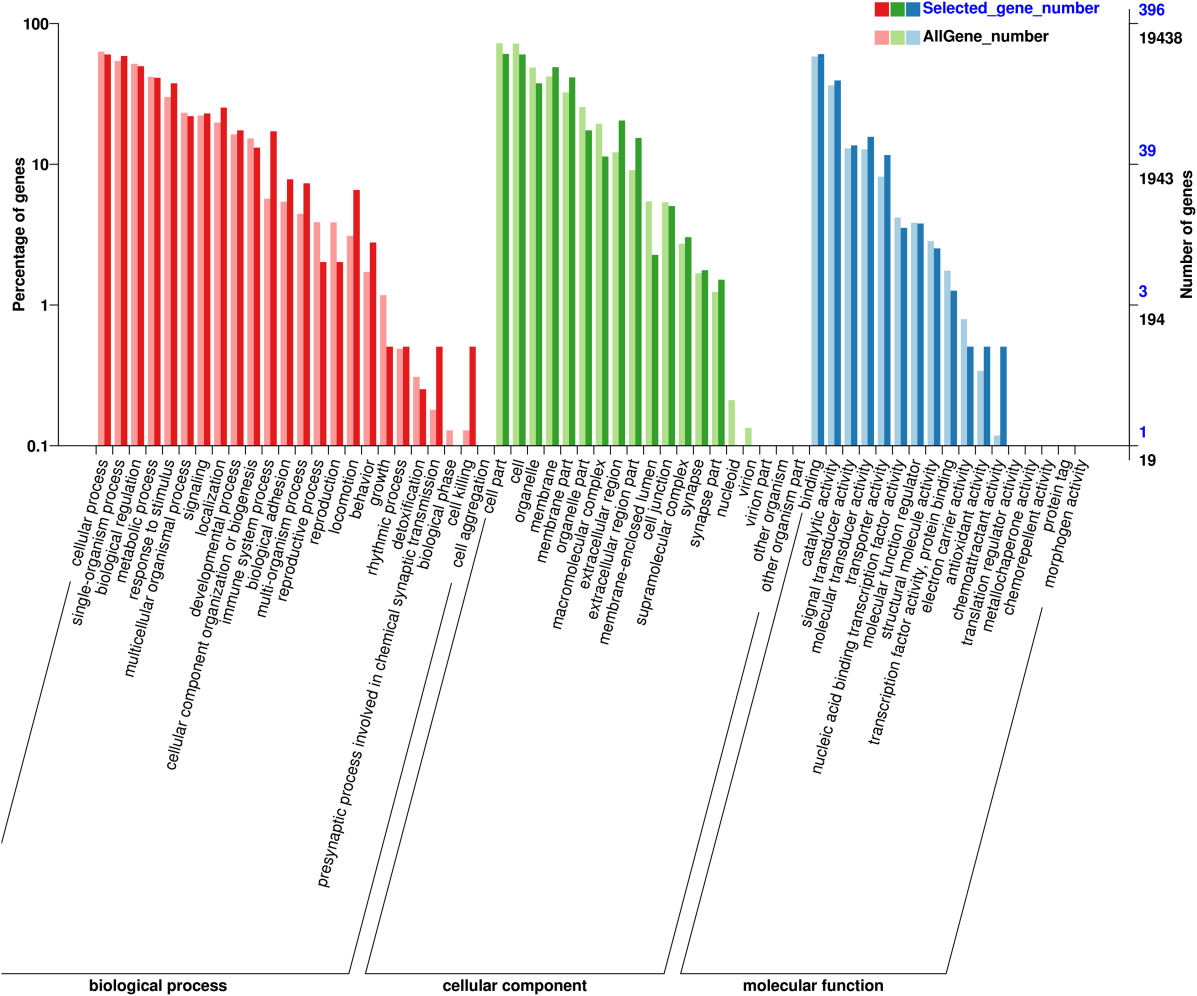
GO analysis of differentially expressed genes. The abscissa is the GO classification, left ordinate is the percentage of the number of genes and right ordinate is the number of genes. This figure shows the gene enrichment of each secondary function of GO in the background of differentially expressed genes and of all genes, reflecting the status of each secondary function in the two backgrounds. The difference in the proportion of secondary functions indicates that the enrichment trend of differentially expressed genes differs from that of all other genes.

GO annotation analysis was performed on differentially expressed genes in the four groups and GO entries with corresponding gene numbers >2 in the three classifications were screened, including 23 biological processes (BP), 14 cellular components (CC) and 12 molecular functions (MF). In the BP functional classification, the annotated genes were mainly classified into cellular process, single-organism process and biological regulation. In the CC function classification, most annotated genes were mainly classified into cell, cell part and organelle; in the MF function classification, most annotated genes were classified as having binding activity, catalytic activity and signal transducer activity (Fig. 2). Among the three comparison groups, coat color genes were routinely classified into some general functions: melanocyte differentiation (GO:0030318), melanosome transport (GO:0032402), developmental pigmentation (GO:0048066), melanin biosynthetic process (GO:0042438) and melanosome (GO:0042470). The expression levels of six of the aforementioned coat color genes were detected in sheep skin in the present study.

**Figure 3:**
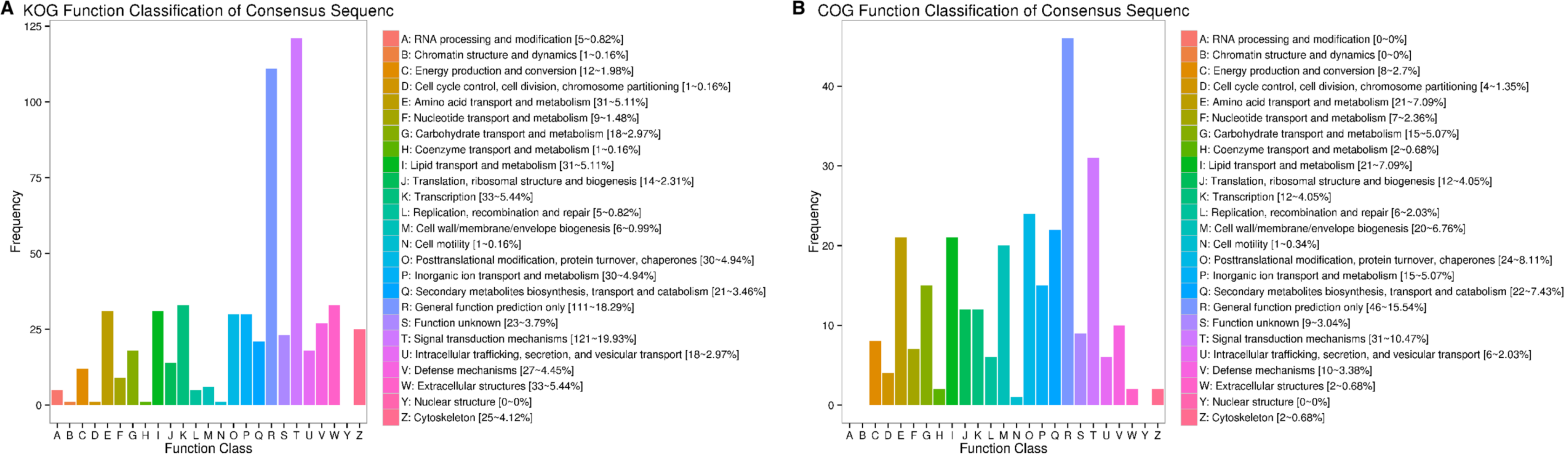
KOG and COG analysis of differentially expressed genes. (A) KOG analysis of differentially expressed genes, (B) COG analysis of differentially expressed genes.

KOG annotation analysis of comparative groups of differential expression genes (Fig. 3A) showed that the main functions of homologous proteins of differentially expressed genes were concentrated in T: signal conduction mechanism (121–19.93%); R: general function (111–18.29%); and K: Transciption (33–5.44%). COG annotation analysis of differentially expressed genes between groups (Fig. 3B) showed that gene expression protein function was mainly concentrated in R: general function (46–15.54%); T: signal conduction mechanism (31–10.47%); and O: Posttranslational modification (24–8.11%).

**Figure 4:**
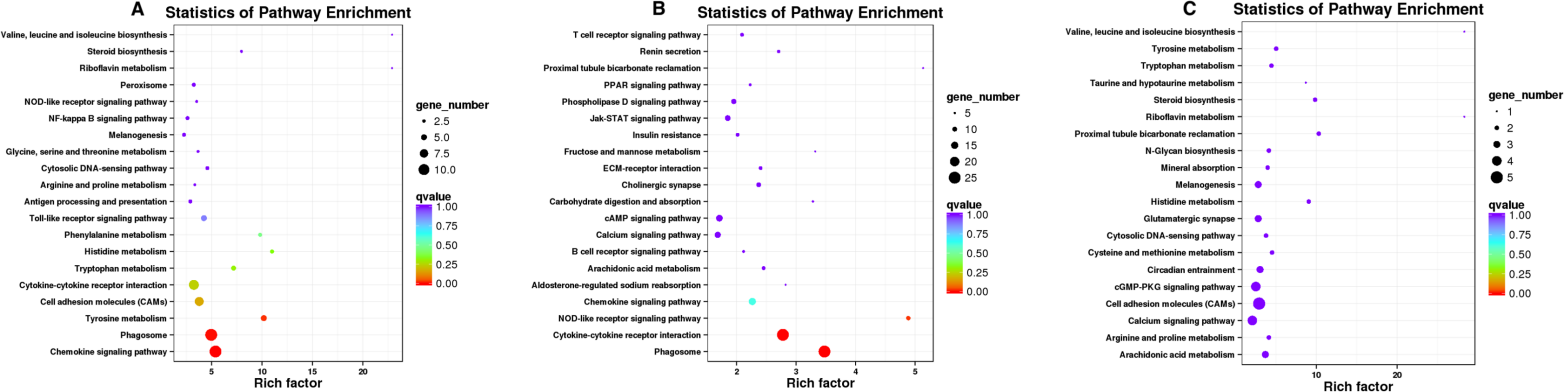
KEGG analysis of differentially expressed genes. (A) W_vs_G, (B) W_vs_R, (C) W_vs_B. Each circle in the figure represents a KEGG pathway and the vertical coordinate represents the name of the pathway while the horizontal coordinate is the enrichment factor, which is the ratio of the number of genes annotated to a certain pathway in differential genes and number of genes annotated to the pathway in all genes. A greater enrichment factor indicates a more significant enrichment level of differentially expressed genes in this pathway. The color of the circle represents *q*-value and the *q*-value is *p* value after multiple hypothesis testing and correction. A smaller *q*-value indicates a more reliable is the enrichment significance of differentially expressed genes in this pathway. The size of the circle indicates the number of genes enriched in the pathway and a larger circle indicates a greater number of genes.

KEGG annotation analysis of differentially expressed genes between groups (Fig. 4) showed that the differentially expressed genes were significantly enriched in pathways associated with immunomodulation, chemokine, cell adhesion molecules, Toll-like receptor, NF-*κ* B, melanogenesis and melanin metabolism and *TYR* metabolism and finally, six differentially expressed genes related to coat color generation including *DCT*, *TYR*, *TYRP1*, *PMEL*, *SLC45A2* and *MLANA*, were identified.

### Relative quantitative real-time RT-PCR

**Figure 5:**
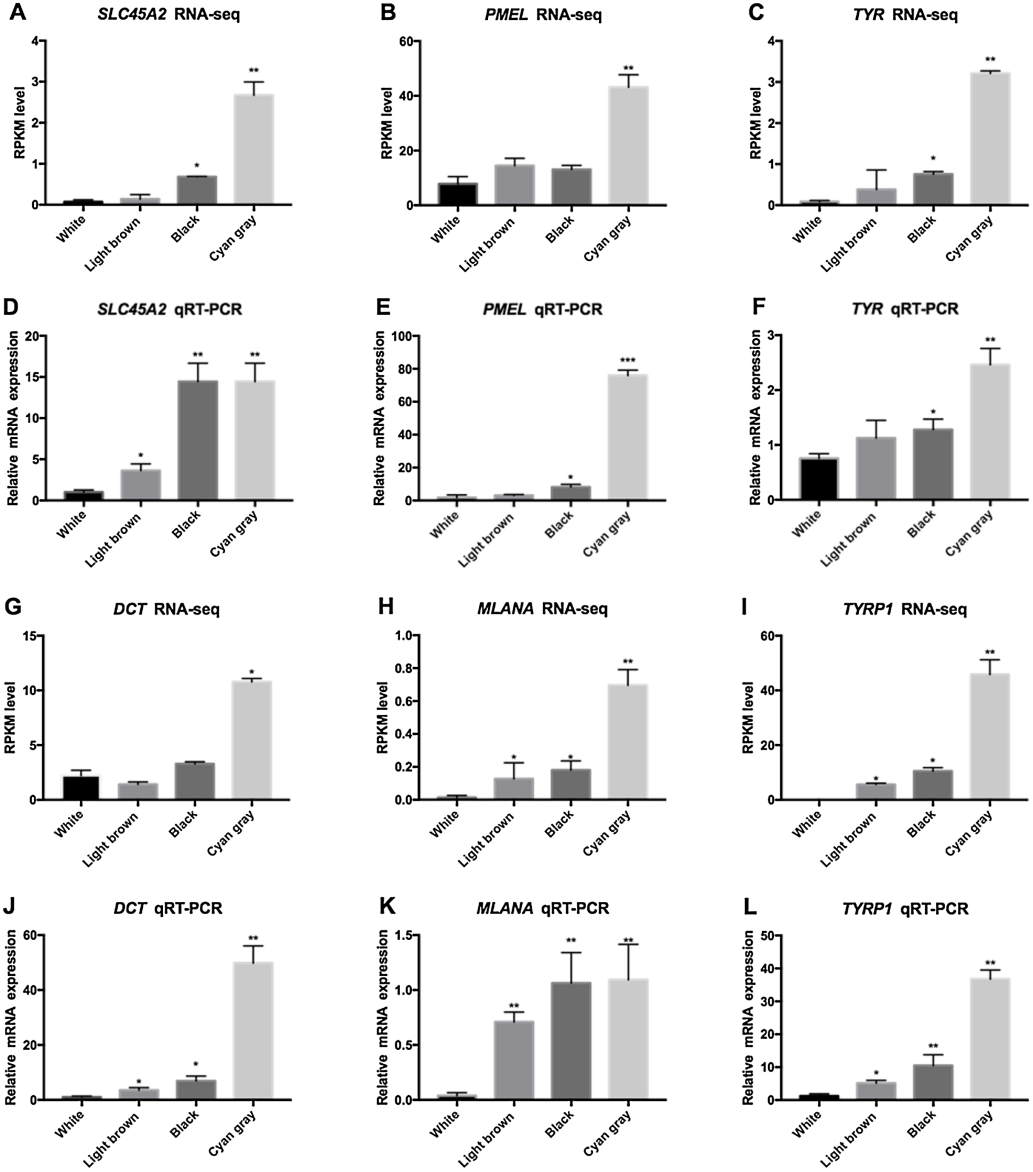
DEGS of qRT-PCR verification analysis. (A–C, G–I) The FPKM values for the six genes selected for this study. The FPKM values are indicated on the y-axis, and gene names are indicated on the x-axis. (D–F, J–L) The qRT-PCR for the 6 genes selected for this study. The Relative expression of six genes on the y-axis, and gene names are indicated on the x-axis.

Six color-related differentially expressed genes (*DCT*, *TYR*, *TYRP1*, *PMEL*, *SLC45A2*, *MLANA*) were screened for gene expression validation in the four sheep groups by real-time fluorescent quantitative PCR (Fig. 5). The results showed that the expression of these six genes in the cyan gray group was higher than that in the black group and the expression trend was consistent with the sequencing results of the transcriptome, indicating the reliability of the sequencing results.

## Discussion

Most mammals have two or more coat colors, which are determined by two pigments on the skin surface. Coat color is an important phenotype in sheep, as it can affect the economic value of the wool. Melanin is produced by various proteins during melanosome development. Melanosomes are organelles in melanocytes that can synthesize and transport melanin (Cieslak et al., 2011).

The premelanosome protein (*PMEL*) is a precursor protein of melanin and important component of the small-molecular weight melanin. It is closely related to the production and deposition of melanin and plays an important role in the synthesis of melanin. Previous studies have shown that *PMEL* gene was related to the silver phenotype. In *Bos taurus* (cattle) with plateaued light-colors, *PMEL* mutation affects melanin synthesis and plays a semi-dominant role in color determination (Schmutz & Dreger, 2013). Two mutations in the *PMEL* gene cause the silvery phenotype of the hourse *Equus caballus* (Sevane, Sanz & Dunner, 2019) and may cause the plumage of *Gallus gallus* to be smoky cyan gray or white (Kerje et al., 2004). Deactivation of *PMEL* in mice caused a large reduction in eumelanin in hair, which plays an important role in maintaining epidermal pigment deposition, but only slightly affected the hair color phenotype of mice (Hellström et al., 2011). In this study, the *PMEL* gene was enriched in the cellular component, melanosome; and in the two BP, melanosome organization and melanin biosynthetic process. This further indicates that the *PMEL* gene is a component of melanosomes in the skin of 1-year-old sheep and also participates in the synthesis of melanin. In addition, quantitative real-time PCR (qRT-PCR) verification found that the expression levels of the *PMEL* gene were up-regulated in the cyan gray group compared with those in the white group but showed no difference between the W_vs_B and W_vs_R groups. This result is similar to observations in Pashmina goats—black, white and brown (Bhat et al., 2019).

An important group of closely related genes that regulate melanogenesis is the tyrosinase gene family (*TYR*, tyrosinase-related protein 1, *TYRP1*, and dopachrome tautomerase *DCT*). *TYR* is the key enzyme for regulation of melanogenesis. *TYR*, *TYRP1* and *DCT* function in stage III of melanosome development, while the melanin corpuscle protein gene, *PMEL*, is one of the major candidate genes that control coat color (Suzuki, 2013). Dopaquinone is a highly active intermediate cycled through a series of complex redox reactions to finally form melanin (Ito, 2006). When *TYR* activity is high, eumelanin is synthesized. In contrast, pheomelanin is synthesized when the activity is not high (Gunnarsson et al., 2007; Nadeau, Burke & Mundy, 2007). The missense mutation His214Asn in *TYR* may induce changes in the chocolate color and melanosome structure in chicken (Ko et al., 2012). Deletion of the 3′ untranslated region of the *TYR* gene in rabbits resulted in reduced melanin synthesis (Sun et al., 2018).

In our study, the *TYR, TYRP1* and *DCT* genes were enriched in the cellular component, melanosome membrane; *TYR and TYRP1* genes were enriched in the BP, melanin biosynthetic process, melanosome organization, and pigmentation; and the *DCT* gene was enriched in the BP, developmental pigmentation and melanin biosynthetic process from *TYR*. qRT-PCR verification found that the expression levels of the *TYRP1* gene were up-regulated in the three comparison groups, consistent with studies in the black and white mink (Song et al., 2017). The expression levels of the *TYR* gene were higher in the cyan gray and black groups than in the white group, which was consistent with studies in chicken (Zhang et al., 2015), but showed no significant differences between the light brown and white groups, consistent with the results of RNA-seq. However, the expression of the *DCT* gene was merely up-regulated in the cyan gray group, consistent with studies in mink and rabbits (Qin et al., 2016; Song et al., 2017).

Solute carrier family 45 member 2 (*SLC45A2*, also known as *MATP*) is a transport-related protein, which plays a role in the classification of melanocyte vesicles and in regulating melanin synthesis (Newton et al., 2001; Inagaki et al., 2006). The proteins encoded by this gene are involved in the processing and transportation of *TYR*, *TYRP1* and *TYRP2* in cells. Missense mutations can also cause albinism in dogs (Wijesena & Schmutz, 2015). An exon four mutation of *SLC45A2* gene may be related to pearl coat color (Sevane, Sanz & Dunner, 2019). In our study, the *SLC45A2* gene was enriched in the cellular component, melanosome membrane; and in the two BP, melanin biosynthetic process and developmental pigmentation. The expression levels of the *SLC45A2* gene were up-regulated in the cyan gray and black groups, which was consistent with studies in small-tailed Han sheep (Wang et al., 2016) and with the results of our RNA-seq analysis.

Melan-A (*MLANA*) participates in the production of melanosomes by maintaining the stability of GPR143, which is crucial for the expression, stability, transport and processing of the melanocyte protein *PMEL*. Together with *PMEL*, it is regulated by the transcription factor *MITF* and up-regulation or down-regulation of *MITF* can regulate endogenous *PMEL* and *MLANA* in melanoma cells. Expression of the *MLANA* gene can also be detected in the skin of patients with vitiligo. In this study, the *MLANA* gene was merely enriched in the cellular component, melanosome. Its expression level in the four groups of coat color was relatively low, although it was up-regulated in the three comparison groups, consistent with the results of RNA-seq.

The six aforementioned color-related differentially expressed genes showed similar expression trends in the four groups of coat color, even though they have different functions. The *PMEL* and *MLANA* genes are components of melanosomes. *PMEL* is involved in melanosome structure determination and acts as a scaffold in melanosomes by forming a proteolytic fibrillar matrix wherein melanin is deposited. Melanosome formation can be subdivided into different stages of maturity, wherein the *PMEL* and *MLANA* genes play a role. Among the four groups of coat color, the expression level of the *PMEL* gene was higher than that of the *MLANA* gene, suggesting that the *PMEL* gene plays a major role in mature melanosomes. Four genes (*DCT*, *TYR*, *TYRP1* and *SLC45A2*) were components of the melanosome membrane. Melanosome membrane channels play an important role in melanin synthesis and thus are also involved in melanin synthesis and transportation. The expression level of the four genes (*DCT*, *TYR*, *TYRP1* and *SLC45A2*) was the highest in the cyan gray group, suggesting that genes determining the dark color of coats are not expressed more than those determining the light color of coats, consequently, further investigations are required to confirm the mechanism of light color phenotype.

## Conclusions

In this study, transcriptome sequencing analysis of the four groups of sheep coat color phenotypes (cyan gray, black, light brown and white) revealed that six differentially expressed genes (*DCT*, *TYR*, *TYRP1*, *PMEL*, *SLC45A2*, *MLANA*) are involved in coat color regulation. These genes were associated with melanocyte differentiation, melanosome transport, developmental pigmentation and melanin biosynthetic process. These six genes, exclusively expressed in the sheep skin with four coat colors, are of particular interest for further studies to elucidate their functional roles in coat color regulation.

## Supplemental Information

10.7717/peerj.8077/supp-1 Supplemental Information 1 Base sequencing error rate distribution. Horizontal coordinate is the base location of reads and vertical coordinate is the single-base error rate.

10.7717/peerj.8077/supp-2 Supplemental Information 2 ATGC content distribution map. Horizontal coordinate is the base location of reads and vertical coordinate is the proportion of a single base.

10.7717/peerj.8077/supp-3 Supplemental Information 3 Distribution of reads on different original parts. Horizontal coordinate is the base location of reads and vertical coordinate is the proportion of a single base.

10.7717/peerj.8077/supp-4 Supplemental Information 4 FPKM box line diagram for each sample.

10.7717/peerj.8077/supp-5 Supplemental Information 5 Differentially expressed gene clustering map. The abscissa represents the sample name and clustering result of the sample and the ordinate represents the clustering result of the differentially expressed genes.

10.7717/peerj.8077/supp-6 Supplemental Information 6 The correlation analysis between each sample.

10.7717/peerj.8077/supp-7 Supplemental Information 7 The primer information of qRT-PCR.

10.7717/peerj.8077/supp-8 Supplemental Information 8 Quality evaluation of sequencing data.

10.7717/peerj.8077/supp-9 Supplemental Information 9 Raw analysis data.

## Additional Information and Declarations

### DNA Deposition

The following information was supplied regarding the deposition of DNA sequences: The sequences are available at NCBI and Figshare. Black group: SRR10143190, SRR10143189, SRR10143186; Cyan gray group: SRR10143185, SRR10143184, SRR10143183; Light brown group: SRR10143182, SRR10143181, SRR10143180; White group: SRR10143188, SRR10143187, SRR10143179. Lidan, Yao (2019): different coat color sheep skin. figshare. Dataset. https://doi.org/10.6084/m9.figshare.8856698.v1.

### Data Availability

The following information was supplied regarding data availability: Raw data is available as a Supplemental File.
