# Supplementary figures and images for: Transcriptome profiling analysis reveals key genes of different coat color in sheep skin
Source: PeerJ. 2019 Nov 21;7:e8077. doi: 10.7717/peerj.8077 (PMC6875393; doi:10.7717/peerj.8077)

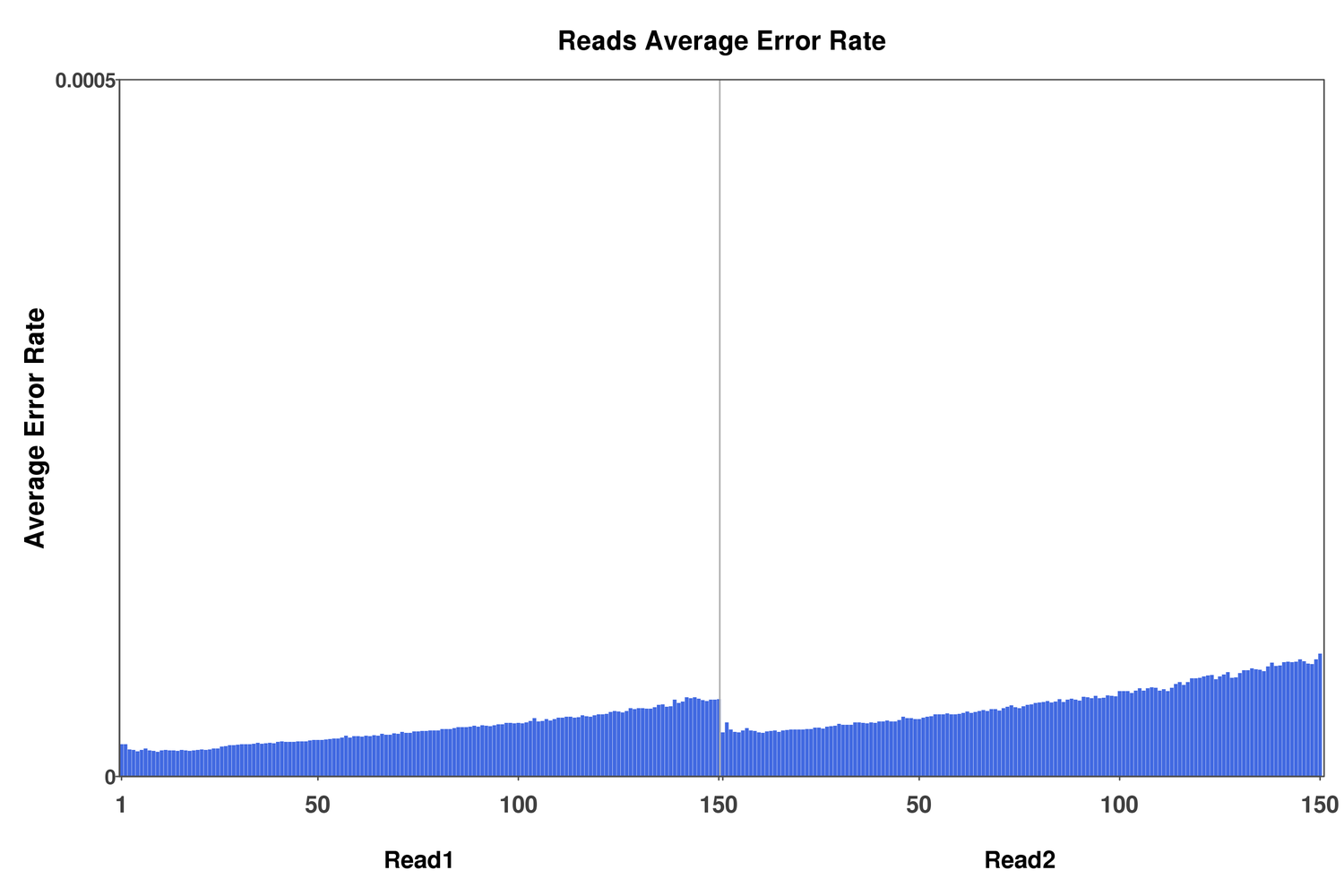

Supplement: Supplemental Information 1 — Horizontal coordinate is the base location of reads and vertical coordinate is the single-base error rate. [file peerj-07-8077-s001.png]

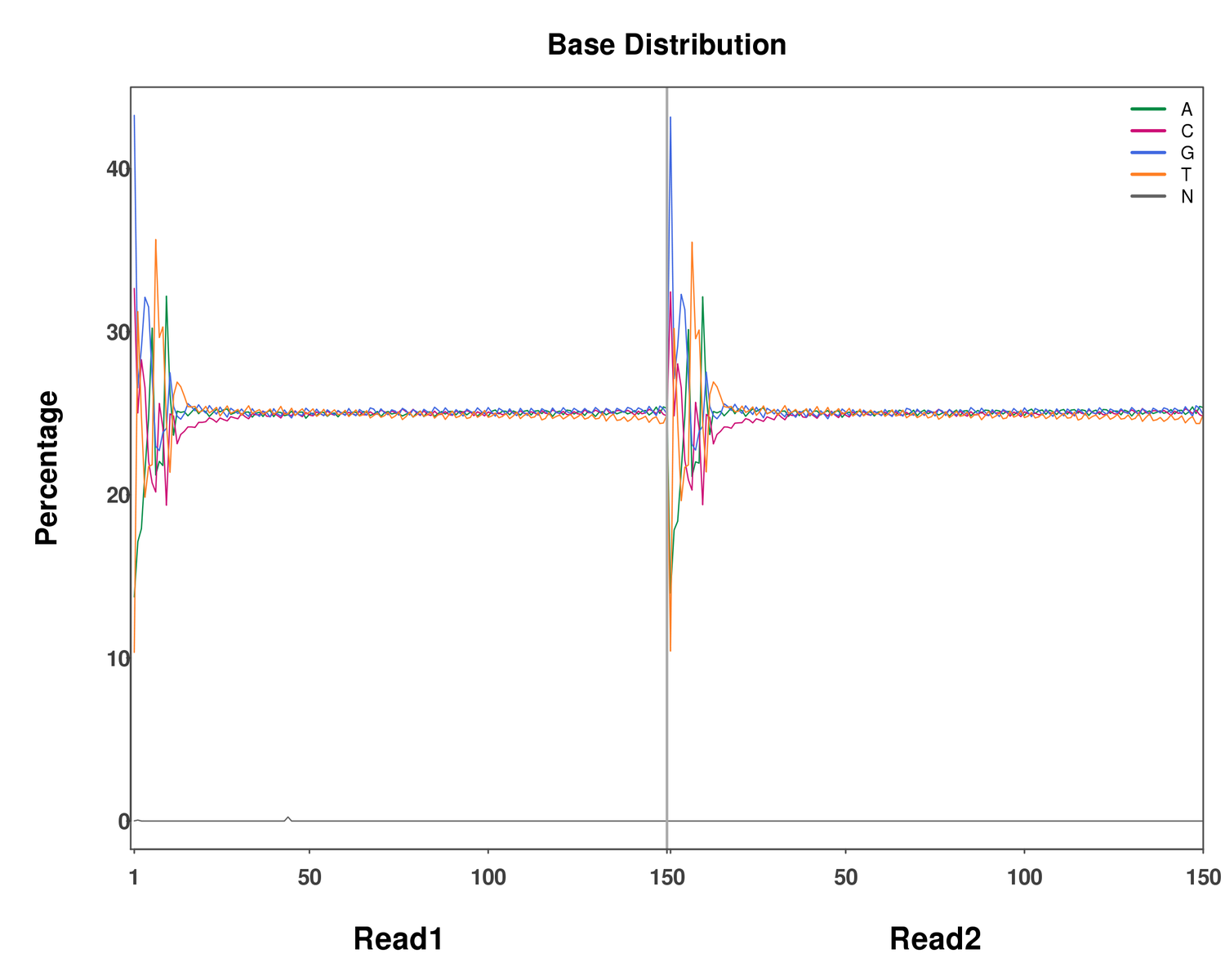

Supplement: Supplemental Information 2 — Horizontal coordinate is the base location of reads and vertical coordinate is the proportion of a single base. [file peerj-07-8077-s002.png]

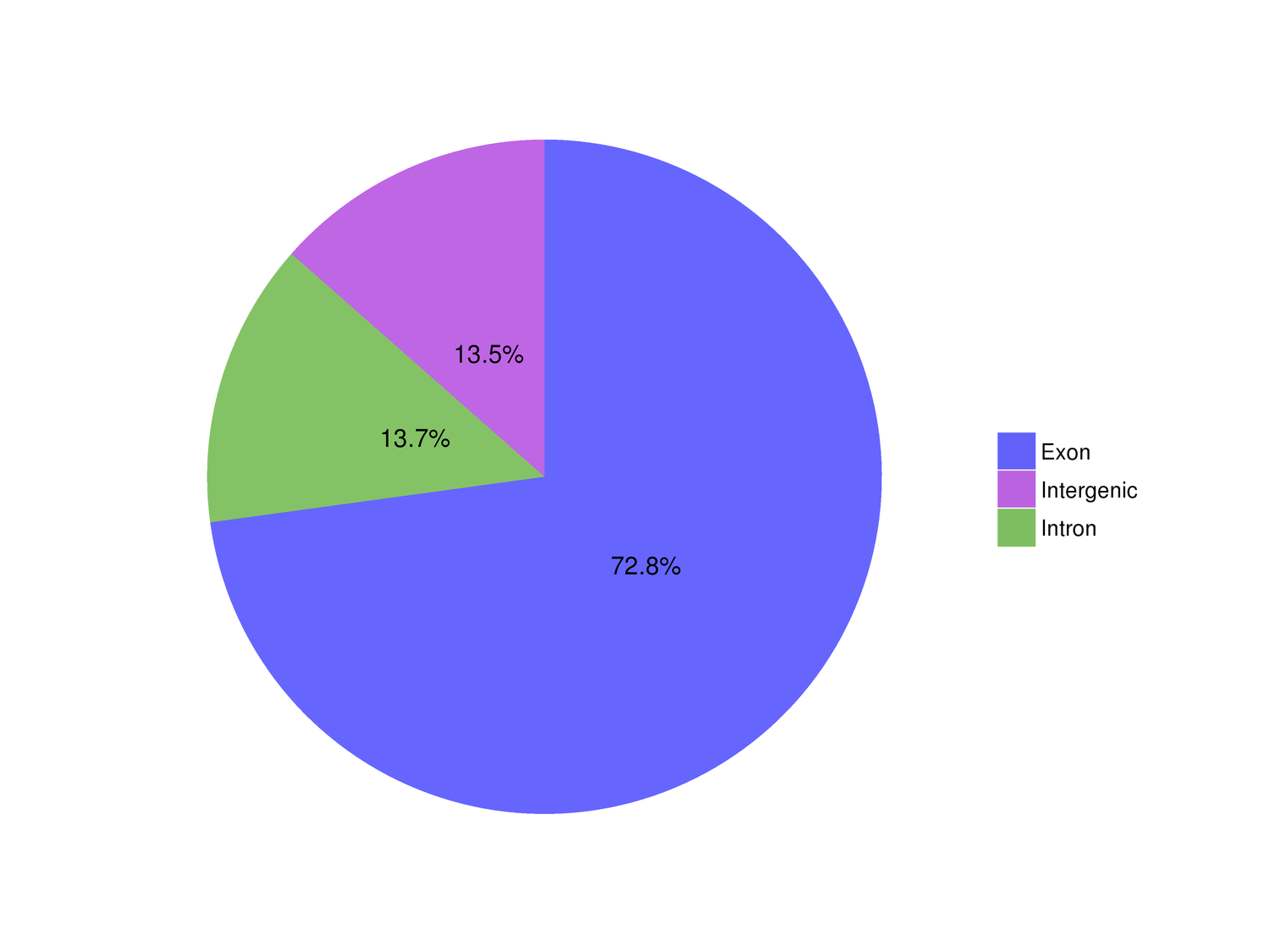

Supplement: Supplemental Information 3 — Horizontal coordinate is the base location of reads and vertical coordinate is the proportion of a single base. [file peerj-07-8077-s003.png]

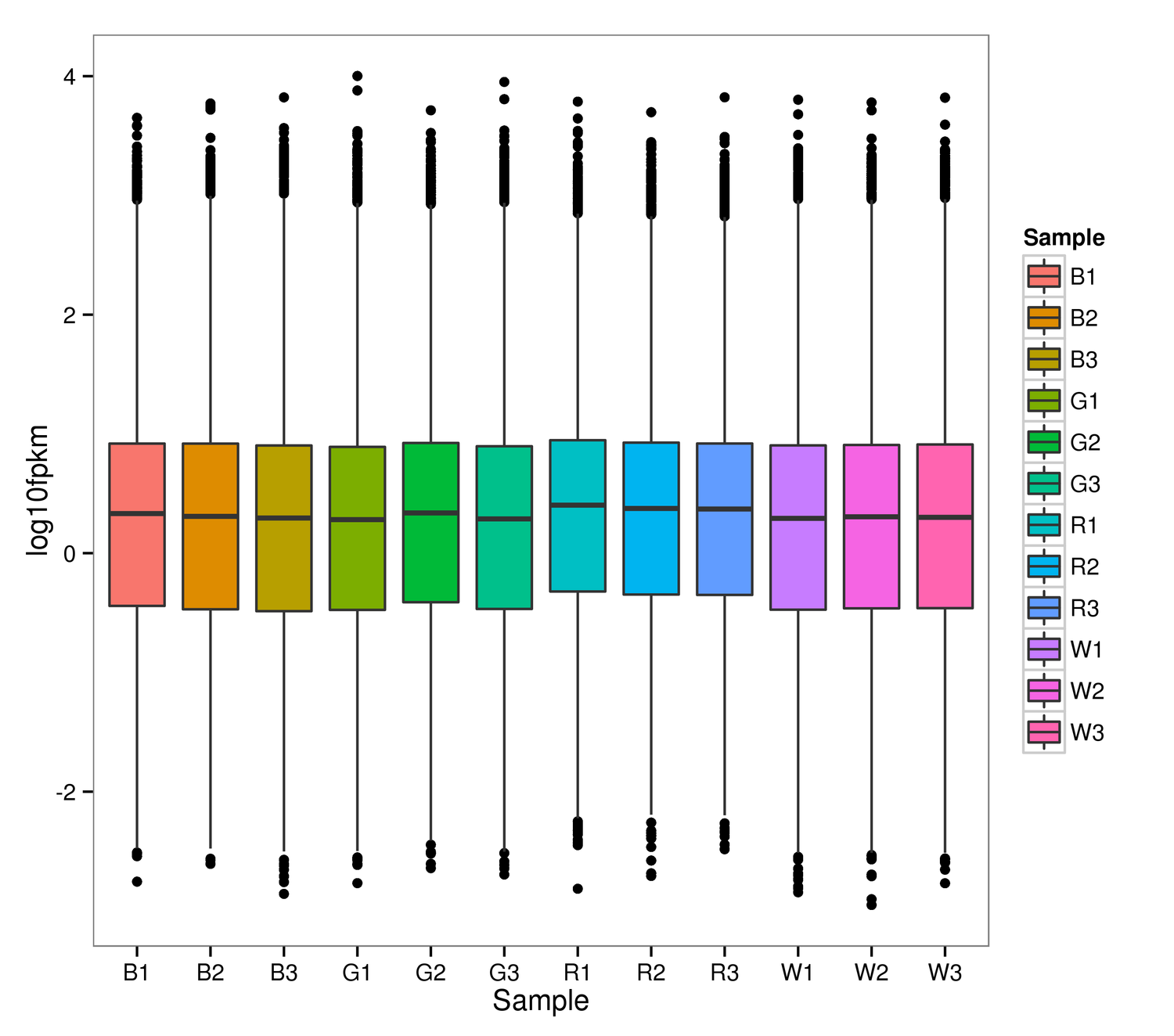

Supplement: Supplemental Information 4 [file peerj-07-8077-s004.png]

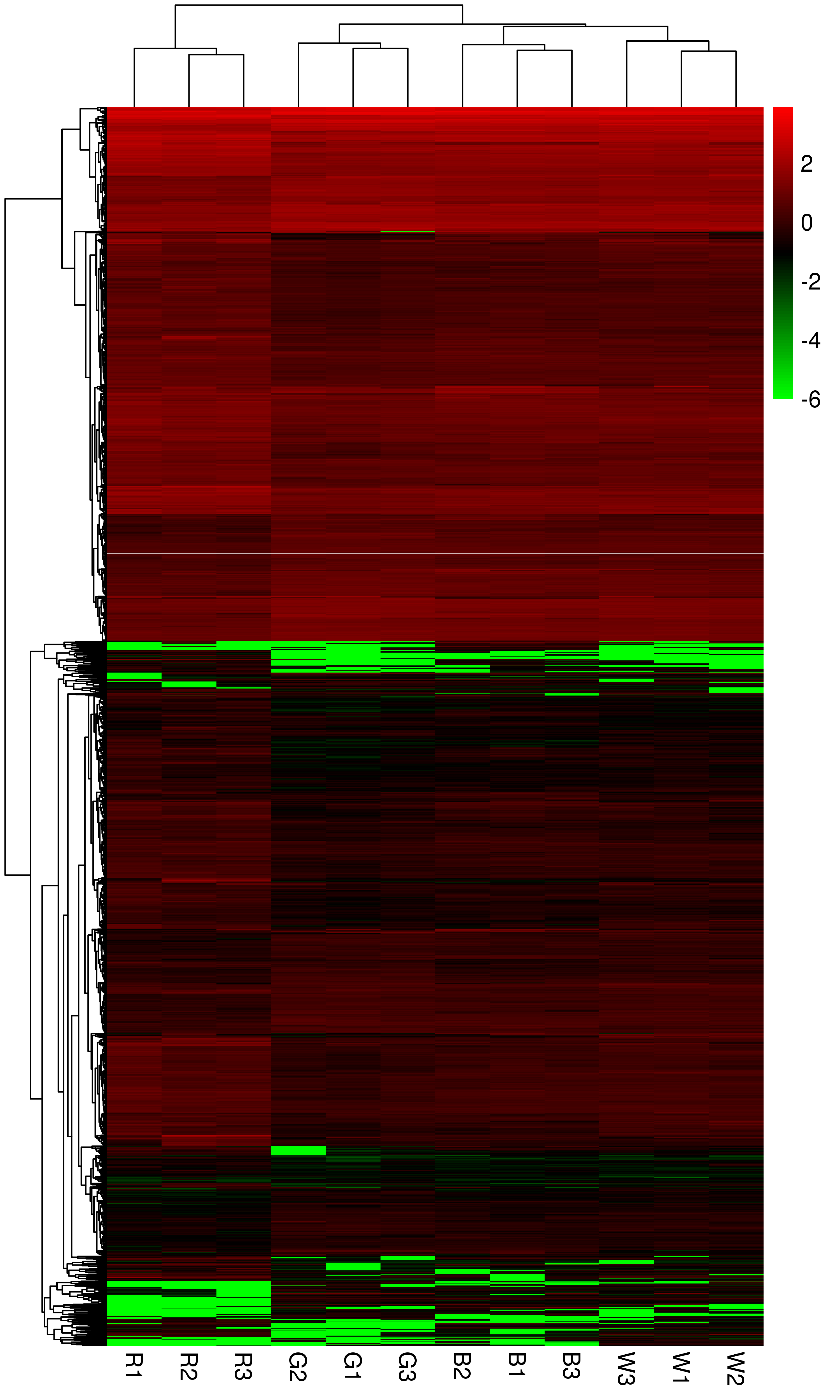

Supplement: Supplemental Information 5 — The abscissa represents the sample name and clustering result of the sample and the ordinate represents the clustering result of the differentially expressed genes. [file peerj-07-8077-s005.png]

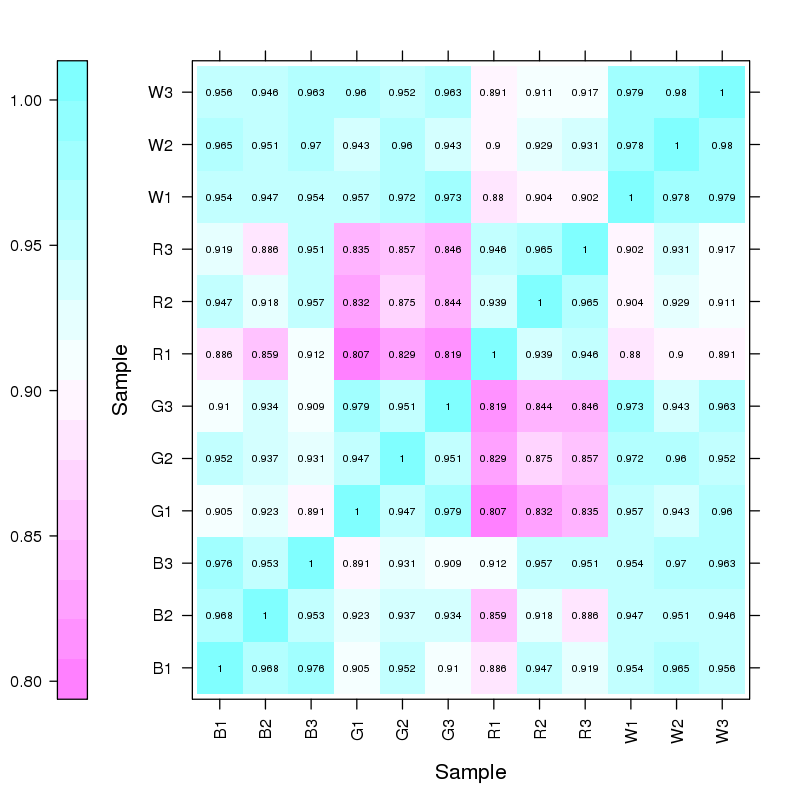

Supplement: Supplemental Information 6 [file peerj-07-8077-s006.png]
