# Supplementary material for: Transcriptome profiling analysis reveals key genes of different coat color in sheep skin
Source: PeerJ. 2019 Nov 21;7:e8077. doi: 10.7717/peerj.8077 (PMC6875393; doi:10.7717/peerj.8077)
Supplement: Supplemental Information 7 [file peerj-07-8077-s007.docx]

| Gene | Primers Sequence(5’-3’) | Product（bp） | Annealing Temp（℃） | Genebank number |
| --- | --- | --- | --- | --- |
| TYR | F:GCGGAAGTTGTAAGTTTGG  R:TCATTTGGCCATAGGTGCCC | 181 | 54 | [NM_001130027.1](https://www.ncbi.nlm.nih.gov/entrez/viewer.fcgi?db=nucleotide&id=194097429) |
| TYRP1 | F:TCACGAGGGACCAGCATTTC  R:CATCGGTGCAAATGTCGCAG | 149 | 51 | [NM_001130023.1](https://www.ncbi.nlm.nih.gov/entrez/viewer.fcgi?db=nucleotide&id=194097412) |
| SLC45A2 | F:ACATTCCCTCACAGCAAGCC  R:CTCCTCAATGCCCTCAACAGT | 193 | 58 | [XM_012163537.1](https://www.ncbi.nlm.nih.gov/entrez/viewer.fcgi?db=nucleotide&id=803321422) |
| DCT | F:CTGGCAATGAGTCCTTCG  R:TCGGCGGTTGTAGTCATT | 182 | 56 | [NM_001130024.1](https://www.ncbi.nlm.nih.gov/entrez/viewer.fcgi?db=nucleotide&id=194097420) |
| MLANA | F:GTCCCTGCTGCTAGGTGTC  R:CCAATCCCTGCAGCCTCTT | 187 | 55 | [XM_015108776.1](https://www.ncbi.nlm.nih.gov/entrez/viewer.fcgi?db=nucleotide&id=965863979) |
| PMEL | F:GAGGCATTGGCACATCTA  R:GGAGCACGGGAATCATAG | 125 | 57 | [XM_012160727.2](https://www.ncbi.nlm.nih.gov/entrez/viewer.fcgi?db=nucleotide&id=965898220) |
| GAPDH | F:GTCCGTTGTGGATCTGACCT  R:GGAGACAACCTGGTCCTCAG | 130 | 52 | [NM_001190390.1](https://www.ncbi.nlm.nih.gov/entrez/viewer.fcgi?db=nucleotide&id=298676424) |
