# Supplementary material for: Transcriptome profiling analysis reveals key genes of different coat color in sheep skin
Source: PeerJ. 2019 Nov 21;7:e8077. doi: 10.7717/peerj.8077 (PMC6875393; doi:10.7717/peerj.8077)
Supplement: Supplemental Information 8 [file peerj-07-8077-s008.docx]

Table S1 Quality evaluation of sequencing data

| Sample Number | Clean reads | Clean bases | GC Content | %≥Q30 | Total Reads | Mapped Reads |
| --- | --- | --- | --- | --- | --- | --- |
| B1 | 29,728,385 | 8,880,602,426 | 51.12% | 94.90% | 59,456,770 | 53,680,573 (90.29%) |
| B2 | 38,524,459 | 11,494,069,436 | 51.45% | 94.92% | 77,048,918 | 69,383,588 (90.05%) |
| B3 | 46,141,873 | 13,765,257,852 | 51.12% | 95.10% | 92,283,746 | 83,336,927 (90.31%) |
| G1 | 36,761,573 | 10,967,820,556 | 52.25% | 94.75% | 73,523,146 | 65,941,101 (89.69%) |
| G2 | 30,386,399 | 9,090,413,472 | 51.28% | 94.21% | 60,772,798 | 54,601,902 (89.85%) |
| G3 | 43,351,590 | 12,916,625,910 | 52.14% | 94.97% | 86,703,180 | 78,088,229 (90.06%) |
| W1 | 45,089,767 | 13,454,768,334 | 51.97% | 94.91% | 90,179,534 | 81,093,677 (89.92%)  1,093,677 (89.92%) |
| W2 | 37,543,106 | 11,207,520,422 | 51.35% | 94.89% | 75,086,212 | 67,676,723 (90.13%) |
| W3 | 36,292,963 | 10,827,195,544 | 51.98% | 94.75% | 72,585,926 | 65,187,666 (89.81%) |
| R1 | 22,020,697 | 6,572,791,648 | 50.78% | 94.25% | 44,041,394 | 39,471,844 (89.62%) |
| R2 | 23,125,125 | 6,906,665,644 | 50.28% | 94.38% | 46,250,250 | 40,965,190 (88.57%) |
| R3 | 23,144,201 | 6,910,463,240 | 50.21% | 94.35% | 46,288,402 | 39,951,923 (86.31%) |
